# Spot-Bonding and Full-Bonding Techniques for Fiber Reinforced Composite (FRC) and Metallic Retainers

**DOI:** 10.3390/ijms18102096

**Published:** 2017-10-04

**Authors:** Andrea Scribante, Paola Gandini, Paola Tessera, Pekka K. Vallittu, Lippo Lassila, Maria Francesca Sfondrini

**Affiliations:** 1 Unit of Orthodontics and Paediatric Dentistry, Section of Dentistry, Department of Clinical, Surgical, Diagnostic and Paediatric Sciences, University of Pavia, 27100 Pavia, Italy; paola.gandini@unipv.it (P.G.); paola.tessera01@universitadipavia.it (P.T.); francesca.sfondrini@unipv.it (M.F.S.); 2 Department of Biomaterial Science and Turku Clinical Biomaterials Centre—TCBC, Institute of Dentistry, University of Turku, 20100 Turku, Finland; pekka.vallittu@utu.fi (P.K.V.); lippo.lassila@utu.fi (L.L.); 3 Welfare Division, 20100 Turku, Finland

**Keywords:** dentistry, orthodontics, prosthodontics, fiber reinforced composite, FRCs, three-point bending, bend, strength

## Abstract

Fiber reinforced Composite (FRC) retainers have been introduced as an aesthetic alternative to conventional metallic splints, but present high rigidity. The purpose of the present investigation was to evaluate bending and fracture loads of FRC splints bonded with conventional full-coverage of the FRC with a composite compared with an experimental bonding technique with a partial (spot-) resin composite cover. Stainless steel rectangular flat, stainless steel round, and FRC retainers were tested at 0.2 and 0.3 mm deflections and at a maximum load. Both at 0.2 and 0.3 mm deflections, the lowest load required to bend the retainer was recorded for spot-bonded stainless steel flat and round wires and for spot-bonded FRCs, and no significant differences were identified among them. Higher force levels were reported for full-bonded metallic flat and round splints and the highest loads were recorded for full-bonded FRCs. At the maximum load, no significant differences were reported among spot- and full-bonded metallic splints and spot-bonded FRCs. The highest loads were reported for full bonded FRCs. The significant decrease in the rigidity of spot-bonded FRC splints if compared with full-bonded retainers suggests further tests in order to propose this technique for clinical use, as they allow physiologic tooth movement, thus presumably reducing the risk of ankylosis.

## 1. Introduction

Fiber reinforced composites (FRCs) were introduced in dentistry over 40 years ago. The reinforcement of dental resins with short or long fibers has been described in alternative to the widely used particulate reinforcements [1,2]. FRCs allow a high strength/weight and stiffness/weight if compared with other materials [3]. Firstly, dental composites have been reinforced with polyethylene, carbon, and aramid fibers [4]. Subsequently, glass fibers [5] have been introduced and, more recently, nanofilled glass FRCs [6] have been presented.

FRCs showed meaningful improvements in properties over unreinforced resins, and usually the clinicians found them easy to manipulate and customize [1,2]. Consequently, during the last years, FRCs have been proposed for many clinical applications [7]. Fixed dental prostheses [8,9], root canal anchoring systems [10,11,12], fillings and core-built ups [13,14,15,16], removable devices [17,18], periodontal and trauma splints [19], orthodontic retainers [20], and orthodontic anchorage units [21] have been reported to be realized with FRCs. Even if FRCs’ high stiffness (33 and 44 N under 0.1 and 0.2 mm deflections, respectively) [22] can be useful for prosthodontic uses, this characteristic could be unwanted for splint and retainer purposes. Some studies have demonstrated that FRC splints presented increased deflection values if compared with metallic wires [22] and conventional stainless steel splints [23,24]. Excessive rigidity can be in contrast with physiologic tooth movement, thus increasing the risk of ankylosis [25,26].

The rigidity of an FRC splint is due to composite and fiber characteristics [27] and can be magnified by the FRC application technique. In fact after enamel etching, the tooth is dried and a thin layer of adhesive resin is applied. The FRC retainer is then located on the enamel surface and a small amount of resin paste is placed to cover the entire retainer and then light cured, as per the manufacturer’s instructions [4,20]. The total composite coverage of an FRC retainer is in contrast with conventional stainless steel splint preparation, which allows the retainer to be covered with resin only in correspondence of each tooth. This fabrication design could enhance the structural elasticity of metallic splints, due to the lack of composite coverage in the interproximal zones of the retainer [28]. On the basis of these considerations, a spot-bonding technique, if applied to FRC splint construction, could decrease FRC rigidity, thus increasing similarity with stainless steel mechanical behaviour.

To our knowledge, bending and fracture loads of FRCs have been tested in the literature [29,30,31], but there is no report that has compared FRCs, prepared with the spot- or full-bonding technique.

**Figure 1:**
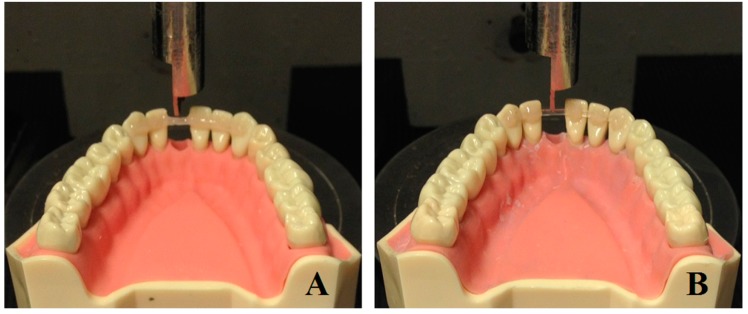
FRC tested with the conventional Full-bond technique (**A**) and with the experimental Spot-bond technique (**B**).

Therefore, the purpose of the present investigation was to evaluate and compare stainless steel (round and rectangular) and FRC splints bonded with full- or spot-composite coverage. The load required to bend the retainer of various deflections was measured (Figure 1).

Strengths were measured at 0.2 and 0.3 mm deflections and at a maximum load (Table 1). The null hypothesis of the present report was that there is no significant difference in deflection values among the various groups tested.

## 2. Results

The descriptive statistics of the loads (N) recorded in the 18 groups including the mean, standard deviation, median, minimum, and maximum are shown in Table 2.

The results of ANOVA indicated significant differences among the various groups (*p* < 0.001).

**Figure 2:**
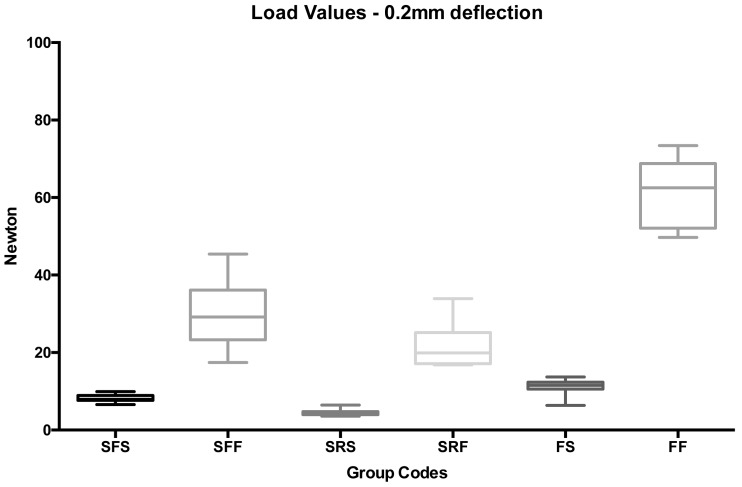
Box plot of load values (N) of the various groups tested at 0.2 mm deflection.

**Figure 3:**
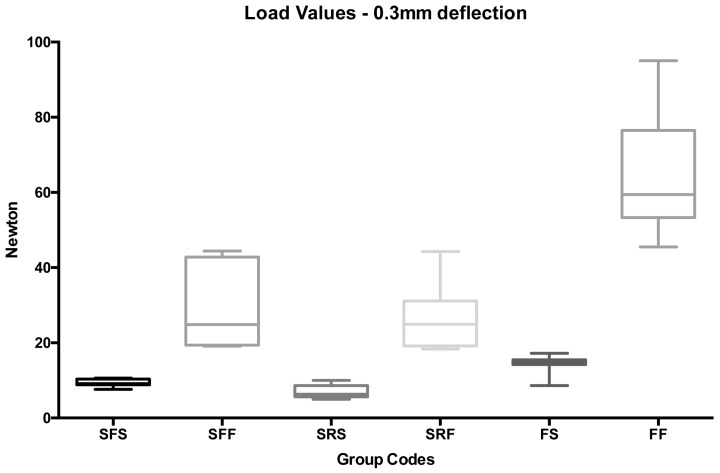
Box plot of load values (N) of the various groups tested at 0.3 mm deflection.

A post-hoc test pointed out that, both at 0.2 mm (Figure 2—groups 1 to 6) and at 0.3 mm (Figure 3—groups 7 to 12) deflections, the lowest strengths were recorded for spot-bonded stainless steel flat (groups 1 and 7) and round (groups 3 and 9) wires and for spot bonded FRCs (groups 5 and 11), and no significant differences were observed among them (*p* < 0.05). Significantly higher force levels were reported for full bonded metallic flat (groups 2 and 8) and round (groups 4 and 9) splints if compared with spot bonded flat (groups 1 and 7) and round (groups 3 and 9) retainers, respectively (*p* < 0.05). The highest strengths were recorded for full bonded FRCs (groups 6 and 12) (*p* < 0.001).

**Figure 4:**
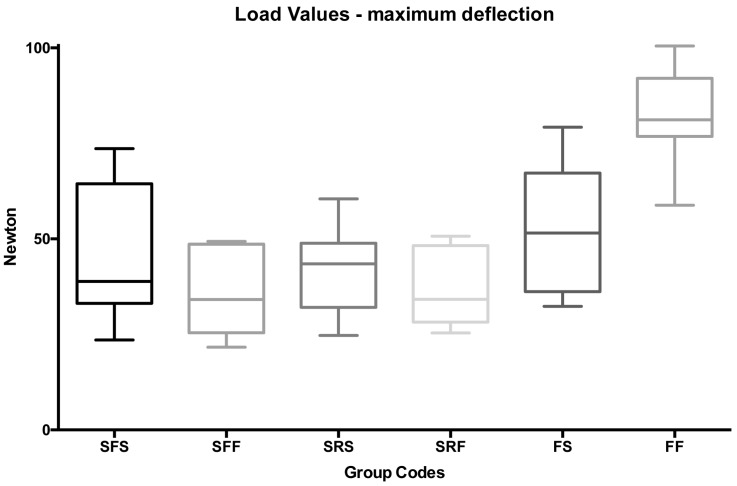
Box plot of load values (N) of the various groups tested at maximum deflection.

On the other hand, at maximum load (Figure 4—groups 13 to 18), no significant differences were reported among spot- and full-bonded metallic flat and round splints (groups 13 to 16) and spot-bonded FRCs (group 17) (*p* > 0.05). Significantly higher loads were reported for full bonded FRCs (group 18) if compared with all other groups tested at maximum deflection (*p* < 0.001).

## 3. Discussion

The null-hypothesis of the study has been rejected. Significant differences in deflection values were reported among the various groups tested.

Full-bonded groups showed significantly higher strength values than spot-bonded groups for flat splints, round splints, and FRCs, at both 0.2 and 0.3 mm deflections. No significant differences among spot-bonded groups (both splints and FRCs) were reported. Therefore, in this study, the spot-bonded technique significantly decreased FRC rigidity, thus allowing a mechanical behaviour similar to flat and round stainless steel splints after 0.2 and 0.3 deflections. A possible reason could be related to the presence of a composite distributed all along the fiber in full bonded groups that could increase the rigidity if compared with the spot-bonded group, in which the composite structure is interrupted between teeth. Another explanation could be related to other variables, such as the internal arrangement of the FRCs. In fact, unidirectional or woven fiber orientation has been reported to influence their mechanical behaviour [3,5,13]. Moreover, the presence of micro- and nano-fillers could also change the FRC characteristics [6]. However, as the spot-bonding technique has not yet been tested, further tests are needed to understand the phenomenon.

Moreover, in the present report, at maximum load, no significant differences between spot and full bonded splints were recorded, whereas significantly higher load values were reported for full-bonded FRCs if compared with spot-bonded FRCs. Therefore, the maximum resistance before the fracture of flat and round splints has been shown to be similar. Maximum load values were reported in the full-bonded FRCs group.

Previous studies have evaluated the load values of conventional and nanofilled FRCs, showing values ranging from 10 to 50 N [6,22,23,30,32,33]. These values are in agreement with the results reported in the present investigation with full-bonded FRCs. To our knowledge, there are no studies that measured the deflection values of FRCs prepared with the spot-bonded technique. In fact, in the literature, previous studies only evaluated spot-bonded metallic splints and full-bonded FRC retainers. To our knowledge, there are no studies that evaluated full-bonded metallic splints. In the present investigation, after 0.2 and 0.3 mm deflections, full-bonded stainless steel retainers showed significantly higher load values than spot-bonded splints (both metallic and FRC) and statistically lower load values than full-bonded FRCs. Therefore, full-bonded metallic splints (both flat and round) exhibited an intermediate mechanical behaviour between spot bonded retainers and full bonded FRCs. Moreover, at maximum load, full-bonded stainless steel retainers showed similar load values to spot-bonded splints (both metallic and FRC) and significantly lower load values than full-bonded FRCs.

The use of multi-stranded spot-bonded wires for the construction of the fixed retainers has been proposed based on their ability to allow the physiological movement of teeth. Moreover, a braided surface offers increased mechanical retention during bonding [25]. Metallic splints presented some disadvantages, mainly related to their aesthetic and the necessity of removal if the patient has to undergo nuclear magnetic resonance exams. Moreover, they cannot be used in patients allergic to metals [20]. For these reasons, FRC retainers have been introduced as a completely aesthetic and metal-free alternative to conventional metallic splints [34]. On the other hand, FRC splints present some disadvantages, in the form of higher costs and the difficulty to repair if debonded [20]. Moreover, the mechanical behaviour of FRC retainers has been reported to be significantly different when compared with metallic ones. Previous studies showed that full-bonded FRC retainers exhibited higher rigidity if compared with metallic wires [22] and splints [23,24,30]. This is in agreement with the present report, as full-bonded FRCs showed significantly higher deflection strengths if compared with flat and round metallic splints.

Some reports showed that the higher rigidity of full-bonded FRC splints could be associated with tooth ankylosis [25,26]. Therefore, the reduction of load values in spot-bonded FRC groups reported in the present investigation could prevent the risk of ankylosis assimilating FRCs behaviour to metallic splints, even if further studies are needed on this topic.

Other studies showed that the clinical durability of an FRC full-bonded splint is over 85% after one year [20,34] and over 65% after two years [35] from bonding. No significant differences were reported between the survival rates of metallic splints and FRC retainers [20,34,35]. These studies support the clinical reliability of full-bonded FRC splints. However, no studies have tested the clinical reliability of spot-bonded FRCs.

When the FRC is left as such in the approximal areas of teeth, oxygen inhibits the free radical polymerization form the surface of the FRC. Therefore, the diameter of the well polymerized FRC is somewhat less than the actual outer diameter of the FRC. The thickness of the oxygen inhibition layer is ca. 0.1 mm which means that the effective diameter (polymerized part of the FRC) of the FRC is not 0.8 mm but ca. 0.6 mm. Such a reduction in the diameter of the retainer causes considerably lower strength and rigidity for the retainer. This may have had an influence on the results with the spot-bonding technique. Therefore, it is advised to add adhesive resin to the surface of an FRC at the approximal areas so that the oxygen inhibition of polymerization occurs in the adhesive rather that in the FRC [36,37].

Bond strengths of full-bonded FRCs have been reported both for new [38] and repaired [39] fibers. Also, the influence of different adhesive systems [40] and polymerization methods [41] has been tested. All these reports showed clinically acceptable bond strength values of conventional full-bonded FRCs, but no studies have been carried out for spot-bonded FRCs.

On the bases of the results of the present investigation, in order to reduce the rigidity of FRC splints, a spot-bonded preparation technique could be proposed. This is the first study that evaluated the spot-bonding technique for FRCs, and in the literature, no other studies have been conducted on such a concern. Therefore, before being routinely used, spot-bonded FRCs should also be tested for other important variables, such as other physical properties, mechanical behaviour, shear bond strength values, biocompatibility, and microbial colonization characteristics.

## 4. Materials and Methods

Rectangular metallic splint wires (Bond-A-Braid, Reliance Orthodontic Products Inc., Itasca, IL, USA), round metallic splint wires (Penta-one 0155, Masel Orthodontics, Carlsbad, CA, USA), and FRCs (Everstick Ortho, StickTech, Turku, Finland) were tested in the present investigation (Table 1).

After a sample size calculation test, all materials were divided into coded groups of 10 specimens each (length: 28 mm), according to different bonding techniques:

-SFS: Stainless steel Flat Spot-bonded
-SFF: Stainless steel Flat Full-bonded
-SRS: Stainless steel Round Spot-bonded
-SRF: Stainless steel Round Full-bonded
-FS: FRC Spot-bonded
-FF: FRC Full-bonded

All specimens were then prepared to be bonded to an acrylic mandible model, simulating a canine-to-canine splint. Element 3.1 was removed from the model before bonding, in order to allow the force to be directly applied to the retainer (Figure 1). The span length between element 3.2 and 4.1 was 8 mm. The two metallic splints (flat and round) and the FRCs were bonded to the elements 3.3, 3.2, 4.1, 4.2 and 4.3 of the mandible model with a one-step, self-etch 7th generation bonding agent (G-aenial Bond, GC America, Alsip, IL, USA) and fixed with flow composite (G-aenial Universal Flo, GC America, Alsip, IL, USA). The composite coverage was complete in the full-bonded splints (Codes: SFS, SRS and FS). In the spot-bonded groups (Codes: SFF, SRF and FF), the composite covered the retainer only in correspondence of each tooth, leaving the splint exposed in interproximal spaces.

All specimens were light-cured (wavelength range of 430–480 nm and light intensity of 1200 mW/cm^2^) by hand with a halogen curing unit (Elipar S10, 3M, Monrovia, CA, USA) for 40 s.

All the stainless steel wires and FRC samples were subsequently tested according to a modified three-point bending test in order to measure the load required to bend the retainer. The load was applied with a universal testing machine (Lloyd LRX; Lloyd Instruments, Fareham, UK) to the middle of the distance between elements 3.2 and 4.1. The strength values were recorded with Nexygen MT software (Lloyd Instruments). The crosshead speed was 1.0 mm per minute [22,32]. Ten specimens for each coded groups were tested at deflections of 0.2 mm (groups 1 to 6), 0.3 mm (groups 7 to 12), and at maximum load (groups 13 to 18). Loads were recorded in newton.

Statistical analysis was performed with a software (R version 3.1.3, R Development Core Team, R Foundation for Statistical Computing, Wien, Austria). Descriptive statistics (mean, standard deviation, minimum, median, maximum, lower confidence interval, and upper confidence interval) were calculated for all the 18 groups tested. The normality of the data was calculated using the Kolmogorov-Smirnov test. As the data were demonstrated to be normal (gaussian distribution), a parametric test was performed. A multi-factor analysis of variance (ANOVA) was performed. Subsequently, a Tukey test was applied as post-hoc, to determine whether there were significant differences among the deflection values of the various groups. Significance for all statistical tests was predetermined at *p* < 0.05.

## 5. Conclusions

The present study demonstrated that both at 0.2 and at 0.3 mm deflections, the lowest loads required to bend the retainer were recorded for spot-bonded stainless steel flat and round wires and for spot-bonded FRCs. Moreover, at maximum load, no significant differences were reported among spot- and full-bonded metallic splints and spot-bonded FRCs.

The significant decrease in the rigidity of spot-bonded FRC splints if compared with full-bonded retainers suggests further tests in order to propose this technique for clinical use.

## Figures and Tables

**Table 1:** Materials tested.

Name	Flat Stainless Steel Wire	Round Stainless Steel Wire	Fiber Reinforced Composite
	Bond-a-Braid	Penta One 0155	FRC Ortho
Manufacturer	Reliance	Masel	StickTech
Material	Stainless steel	Stainless steel	E-glass fiber 15 μm
Dimensions	0.673 mm (w) × 0.268 mm (h)	Diameter: 0.394 mm	Diameter: 0.75 mm
Unit Amount	8 wires	5 wires	1000 fibers
Design	Ribbon arch	Coaxial	Unidirectional fibre bundle

**Table 2:** Descriptive statistics (N) of the load values of the 18 groups tested (each group consisted of 10 specimens).

Group	Code	Material	Shape	Bonding	Deflection (mm)	Mean	SD	Min	Mdn	Max	Lower CI	Upper CI	Post-Hoc *
1	SFS	Stainless steel	Flat	Spot bonded	0.2	8.20	1.03	6.57	7.93	9.94	7.48	9.23	A
2	SFF	Stainless steel	Flat	Full bonded	0.2	30.18	8.91	17.43	29.17	45.41	24.01	39.10	B, I
3	SRS	Stainless steel	Round	Spot bonded	0.2	4.60	0.86	3.60	4.59	6.43	4.00	5.46	A
4	SRF	Stainless steel	Round	Full bonded	0.2	21.79	5.88	16.83	19.90	33.93	17.71	27.67	B
5	FS	FRC	-	Spot bonded	0.2	11.13	2.16	6.37	11.50	13.69	9.63	13.28	A, C
6	FF	FRC	-	Full bonded	0.2	61.70	8.75	49.72	62.53	73.40	55.64	70.45	D, G, H
7	SFS	Stainless steel	Flat	Spot bonded	0.3	9.34	1.00	7.63	9.12	10.59	8.65	10.35	A, C
8	SFF	Stainless steel	Flat	Full bonded	0.3	29.37	11.16	19.12	24.84	44.43	21.64	40.53	B, I
9	SRS	Stainless steel	Round	Spot bonded	0.3	6.89	1.79	4.98	6.30	10.00	5.66	8.68	A
10	SRF	Stainless steel	Round	Full bonded	0.3	26.35	8.74	18.33	24.93	44.29	20.30	35.09	B, C, J
11	FS	FRC	-	Spot bonded	0.3	14.37	2.51	8.62	14.80	17.23	12.63	16.89	A
12	FF	FRC	-	Full bonded	0.3	64.78	16.29	45.52	59.42	95.02	53.49	81.08	D, E, H
13	SFS	Stainless steel	Flat	Spot bonded	Maximum Load	46.44	18.21	23.50	38.88	73.62	33.82	64.64	F, G, I
14	SFF	Stainless steel	Flat	Full bonded	Maximum Load	36.17	11.33	21.66	34.12	49.32	28.32	47.50	F, I, J
15	SRS	Stainless steel	Round	Spot bonded	Maximum Load	41.67	11.40	24.68	43.45	60.47	33.77	53.06	F, I, J
16	SRF	Stainless steel	Round	Full bonded	Maximum Load	37.25	10.00	25.38	34.15	50.71	30.32	47.26	F, I, J
17	FS	FRC	-	Spot bonded	Maximum Load	52.20	16.55	32.30	51.48	79.19	40.74	68.75	F, G
18	FF	FRC	-	Full bonded	Maximum Load	81.86	12.56	58.81	81.15	100.51	73.16	94.42	E

*: Mean with same letters are not significantly different.
